# Simulation of coal resistivity dynamics during methane adsorption and desorption using an electrical rock physics model

**DOI:** 10.1038/s41598-025-09650-3

**Published:** 2025-07-18

**Authors:** Jiaqi Zou, Shuangquan Chen, Yuanji Li, Tingting Yu

**Affiliations:** 1 National Key Laboratory of Petroleum Resources and Engineering, CNPC Key Laboratory of Geophysical Exploration, China University of Petroleum (Beijing), Beijing, 102249 China; 2 Department of Safety Engineering, Heilongjiang University of Science and Technology, Harbin, 150022 China; 3 State Key Laboratory of Continental Shale Oil, Daqing Oilfield Limited Company, Daqing, 163712 China

**Keywords:** Coalbed methane, Electrical rock physics modeling, Adsorption and desorption, Correction factors, Applicability analysis, Geophysics, Mineralogy

## Abstract

Understanding the correlation between coal resistivity and methane content is critical for optimizing coalbed methane (CBM) recovery and ensuring mining safety. Existing studies mainly rely on empirical trend fitting, leaving a gap in model-driven analyses of resistivity dynamics during methane adsorption and desorption. This study develops a dual-coefficient electrical rock physics model integrating inorganic mineral composition, organic resistivity, methane adsorption–desorption behavior, and pore inclusion structures. Correction coefficients (0.2 for methane and 0.4 for organic resistivity) were introduced to address adsorption heterogeneity and structural complexity. Experimental validation on coal samples (density: 1.45 g/cm^3^, porosity: 5.5%) showed strong agreement between simulated and measured resistivity during adsorption (0.8882–3.6973 m^3^/t) and desorption (3.3974–2.1773 m^3^/t), with high correlation (R^2^ = 0.9815 adsorption, 0.9956 desorption; *P*-values = 0.9861, 0.9763). Sensitivity analysis revealed that mineral composition (e.g., quartz, clay) and inclusion aspect ratios (0–1) notably affect resistivity. Flattened inclusions (low aspect ratios) reduce resistivity more than spherical ones, especially at methane volumes lower than 0.15 m^3^/t. Organic content inversely correlates with resistivity; when the volume fraction exceeds 0.92, pore structure effects diminish. This work links microscopic adsorption mechanisms to macroscopic electrical properties, providing a predictive framework for CBM resource evaluation, CO_2_ storage monitoring, and coal mine hazard mitigation. The model adapts to diverse coal types and structural conditions, demonstrating broad applicability in research and industry.

## Introduction

The efficient extraction and safe utilization of coalbed methane (CBM) are critical for advancing clean energy transitions and mitigating coal mine hazards. A key challenge lies in understanding the dynamic relationship between methane adsorption/desorption and coal resistivity, as resistivity serves as a proxy for indirectly assessing gas content^1–3^. Current approaches remain largely empirical, relying on trend-fitting of experimental data rather than mechanistic modeling. Accurate characterization of coal’s resistivity response to methane dynamics is essential for optimizing CBM recovery, predicting gas outburst risks, and evaluating CO_2_ storage potential^4^. Despite its importance, the interaction among coal’s heterogeneous mineralogy, pore structure, and resistivity variations induced by methane remains poorly quantified. This limits the development of predictive tools for field-scale applications.

The Langmuir model is currently regarded as the most appropriate theoretical framework for simulating methane adsorption in coal. The Langmuir volume, obtained by fitting adsorption data, is widely used to describe coal’s methane adsorption capacity^5–8^. Numerous studies have examined the relationship between methane adsorption and coal micropores to better understand the mechanism and predict methane occurrence in coal seams. Most of these studies have focused on specific surface area (SSA)^9–11^, micropore volume^12,13^, and coal particle size distribution (MPSD)^14^, leading to the emergence of two main perspectives. The first perspective holds that a larger SSA provides more adsorption sites, thereby increasing methane adsorption capacity^1^. In this view, adsorption capacity is linearly related to SSA under varying pressure conditions^15,16^. The second perspective suggests that adsorption capacity is more strongly correlated with coal volume^11,13,17^. Lozano-Castelló et al. reported that methane adsorption depends on both coal volume and micropore size distribution (MPSD)^10,12,14^. Mining alters in-situ stress and pore pressure in coal seams, changing the geometry of pores and fractures. These changes influence gas adsorption and diffusion, potentially leading to unanticipated outcomes and safety risks^18^. Tesson and Firoozabadi used molecular dynamics (MD) simulations to investigate methane adsorption in kerogen frameworks with varying surface roughness under different temperatures and pressures^19^. Tol, Mechaev, and Li employed MD methods to analyze gas behavior in fractured coal pore spaces^20,21^. Fang et al. examined coal’s molecular response to stress, observing that increased stress raised cell density while reducing methane adsorption capacity and system energy^22^. Peng et al. identified the main factors controlling adsorption–desorption hysteresis using kinetic simulations^23^. Wang et al. compared methane behavior across different coal types through isothermal adsorption–desorption experiments to assess microstructural changes^24^. Chu et al. quantitatively analyzed the relationship between desorption hysteresis and pore structure parameters^25^.

Previous studies have extensively investigated coal’s methane (CH₄) adsorption capacity, gradually clarifying the mechanisms governing coalbed methane adsorption. Concurrently, variations in methane content directly influence coal’s electrical resistivity. Increasing attention has been directed toward analyzing adsorption and desorption processes through resistivity data. During methane adsorption and desorption, processes such as gas diffusion, seepage, and coal matrix deformation occur simultaneously, all of which are directly or indirectly linked to changes in resistivity^26,27^. Xu et al. reported that conductivity increases with frequency and is influenced by the fractal characteristics of the coal structure^28^. Wang et al. examined resistivity changes under uniaxial compression in methane-free conditions^29^. Li et al. developed a triaxial stress testing system for coal resistivity and, through combined experimental and theoretical analysis, evaluated resistivity anisotropy under different loading paths, providing a theoretical foundation for dynamic disaster prediction in coal mines^30^. Yang further explored the correlation between resistivity variation and methane outburst prediction^31^. Wang et al. assessed the evolution of coal’s electrical parameters during deformation and failure under different pressure conditions^32^. Chen et al. simulated desorption via compression, gas injection, and rapid pressure release, revealing resistivity changes in gas-bearing coal^33^. In a subsequent study, Chen analyzed real-time resistivity changes during methane adsorption and desorption across various coal samples and pressure conditions, elucidating their resistivity patterns and mechanisms^34^. Feng et al. observed that resistivity increases linearly as desorbed gas volume decreases during methane desorption^35^. They also observed that higher adsorption pressures led to greater coal swelling, compressed internal particles, and amplified resistivity changes. Zhu et al. examined volume resistivity during coal spontaneous combustion and developed a temperature-resistivity model. Results indicated that resistivity initially increased and then decreased with rising temperature^36^. Lyu et al. studied CO_2_ adsorption and resistivity changes in raw and molded coal under varying temperatures, offering insights for CO_2_ concentration prediction^37^. Shao et al. measured the resistivity and permeability of various coal rocks at elevated temperatures and identified the causes of resistivity variation^38^.

In summary, numerous scholars have conducted detailed studies on the methane adsorption capacity and the changes in electrical properties due to adsorption in coal. However, two key questions remain to be addressed: (1) How can the equivalent resistivity characteristics of the coal framework be accurately described? (2) How can methane adsorption and desorption under variable pore structure conditions be effectively simulated?

This study addresses these gaps by developing a two-coefficient electrical rock physics model that incorporates inorganic minerals, organics, methane adsorption–desorption behavior, and pore structure heterogeneity. The model introduces three main innovations: First, the electrical Hassing-Strickman (eHS) and differential effective medium (eDEM) theories were combined to simulate resistivity changes during methane adsorption and desorption. A correction factor (0.2 for methane and 0.4 for organics) was applied to account for adsorption heterogeneity. Second, the effects of pore geometry (aspect ratio: 0–1) and mineral composition on resistivity were quantified. The results indicated that flat inclusions reduce resistivity more significantly than spherical pores, especially at methane volumes lower than 0.15. Finally, the model was validated using a coal sample (density: 1.45 g/cm^3^, porosity: 5.5%) with a strong correlation (R^2^ > 0.98) between simulated resistivity and measured resistivity during adsorption (0.8882–3.6973 m^3^/t) and desorption (3.3974–2.1773 m^3^/t). This model provides a mechanistic link between methane behavior and coal’s electrical response, advancing the understanding of rock physics and offering a generalized tool for CBM resource evaluation, carbon monoxide storage assessment, and coal mine safety management.

## Model development

### Modeling workflow

Electrical conductivity in rocks arises from the directional movement of charged particles, primarily free electrons from the mineral framework and ions from pore fluids. Methane adsorption in coal alters the distribution and behavior of these charge carriers, thereby influencing the effective resistivity of the coal. A reliable coal resistivity model must incorporate methane adsorption effects across various pore structures. Coal consists of both organic and inorganic components. The organic matter is composed mainly of carbon, hydrogen, and oxygen, which collectively account for over 95% of its total content, along with smaller amounts of nitrogen and organic sulfur. The inorganic fraction predominantly includes clay, quartz, potassium feldspar, and plagioclase. The pore structure of coal can be broadly classified into inter-mineral pores and micro-fractures within the organic matrix. This pore structure is a critical determinant of coal’s methane adsorption capacity.

**Fig. 1:**
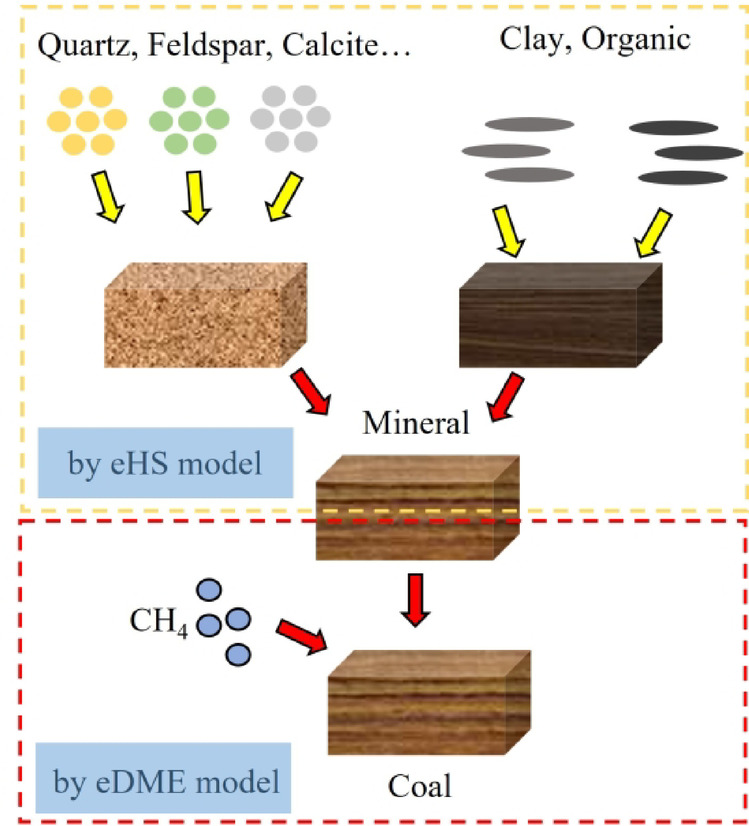
Electrical petrophysical modeling process.

The electrical rock physics modeling process integrates mineral composition, coal matrix structure, and methane effects under variable pore conditions. The overall workflow is illustrated in Fig. 1, and the modeling steps are summarized as follows:

1. *Modeling inorganic mineral components*. High-resistivity minerals such as quartz, feldspar, and calcite are modeled using the eHS model to compute their equivalent resistivity after mixing.
2. *Mixing clay and organic matter*. Given the relatively similar resistivity of clay and organic matter, the eHS model is also applied to combine these components and calculate their combined equivalent resistivity.
3. *Combining the results from steps 1 and 2*. The equivalent resistivities from the previous steps are integrated using the eHS model to represent the overall coal matrix.
4. *Incorporating methane effects*. The eDEM model accounts for methane adsorption and desorption effects, including resistivity changes across different pore geometries.

This section provides an overview of the modeling framework. The rationale behind each step is addressed in the following sections. The proposed method offers a comprehensive approach to simulate coal resistivity as a function of mineral composition and methane interaction within heterogeneous pore structures. Model parameters are defined in the Nomenclature.

### eHS model

Recent studies on the resistivity of multiphase materials have emphasized the roles of phase volume fractions and microstructural characteristics. Various analytical models have been proposed to describe the effective mixing behavior of these phases. From a micromechanical perspective, boundary theories offer a practical approach to modeling composite systems. These theories can yield reliable bounds on material properties even when only limited information, such as volume fraction and macroscopic isotropy, is available. Among these, boundary theories proposed by Wiener^39^ and Hashin-Shtrikman^40^ explicitly delineate the upper and lower bounds of effective conductivity (the inverse of resistivity) in multiphase systems. These bounds remain valid regardless of microstructural complexity, provided the constituent phases are well-characterized^41^. Thus, boundary theories provide key modeling constraints and can be adopted to effectively predict multiphase resistivity without requiring detailed microstructural data.

The HS model was originally developed to estimate the elastic moduli of two-phase media. It assumes spherical inclusions, with one phase embedded within another. Designating the harder material as the host yields the upper bound, whereas assigning the more flexible material as phase as the host yields the lower bound^42^. The model has since been applied to estimate properties in diverse systems, including partially molten rocks, soils, and elastic composites^43–46^. The expressions for the upper ($\sigma_{{{\text{HS}}^{ + } }}$) and lower ($\sigma_{{{\text{HS}}^{ - } }}$) bounds of electrical conductivity are given as follows^47^:

1 $$\sigma_{{{\text{HS}}^{ + } }} = \sigma_{0} + \frac{{\sigma_{0} f_{1} }}{{\frac{{\sigma_{0} }}{{\sigma_{1} - \sigma_{0} }} + \frac{{f_{0} }}{3}}}$$

2 $$\sigma_{{{\text{HS}}^{ - } }} = \sigma_{1} + \frac{{\sigma_{1} f_{0} }}{{\frac{{\sigma_{1} }}{{\sigma_{0} - \sigma_{1} }} + \frac{{f_{1} }}{3}}}$$

where $\sigma_{1}$ and $\sigma_{2}$ denote the electrical conductivities (S/m) of phases 1 and 2, respectively, which are the reciprocals of their resistivities ($\Omega \;{\text{m}}$). Similarly, $f_{1}$ and $f_{2}$ represent the volume fractions of the respective phases. Berryman (1995) extended the HS theory to accommodate multiphase systems^48^. This extension allows for the estimation of effective conductivity (or resistivity) in media composed of more than two components. The method offers a framework for estimating the bounds of electrical properties in complex, multiphase materials based on their volume fractions and the properties of individual phases:

3 $$\sum {(s)} = \left( {\sum\limits_{i = 1}^{N} {\frac{{f_{i} }}{{\sigma_{i} + 2s}}} } \right)^{ - 1} - 2s$$

where *s* is $\sigma_{\min }$ or $\sigma_{\max }$. The term $f_{i}$ corresponds to the volume fraction of each phase within the material.

In cases where only the volume fractions of each phase are known, the HS model provides the most applicable bounds for multiphase resistivity estimation. In coal, aside from the organic fraction, common inorganic minerals include quartz, feldspar, and calcite. The resistivity variation among these minerals is relatively minor compared to the contrast with clay and organic matter. Therefore, HS bounds can be effectively employed to estimate the resistivity contribution of inorganic minerals. Clay and organic matter typically exhibit large volume fractions and low resistivities, often several orders of magnitude lower than those of the inorganic minerals. As a result, these two phases can be treated as a combined low-resistivity component in the modeling process. This approach improves the accuracy of the overall resistivity prediction by accounting for the collective behavior of both low- and high-resistivity phases.

### eDEM model

Maxwell first modeled inclusions as spherical particles and employed an effective medium approximation to describe the electrical properties of composite materials^49^. Bruggeman (1935) extended this approach to account for more complex multiphase systems, forming the basis of the Differential Effective Medium (DEM) model^50^. Polder later introduced a method for calculating average electrical properties in media containing randomly oriented ellipsoidal inclusions, assuming no interaction between inclusions^51^. Frank subsequently derived the effective electric field of a single ellipsoidal inclusion embedded in a host medium^52^. Building on these foundations, Sen et al.^53^, and later Mendelson and Cohen, developed a DEM formulation for effective conductivity in materials containing randomly distributed ellipsoidal inclusions in a uniform electric field^54–56^.

The DEM model captures both the volume fraction and geometric characteristics of inclusions. For practical application, the key expressions are presented below without detailed theoretical derivations. The effective conductivity σ of the mixture is given by:

4 $$\frac{{{\text{d}}\sigma^{*} }}{{\sigma^{*} - \sigma_{2} }} = \frac{1}{3}\sum\limits_{p = 1}^{3} {\langle \left[ {1 + \left( {\frac{{\sigma_{2} }}{{\sigma^{*} }} - 1} \right)L_{p} } \right]^{ - 1} \rangle } \frac{{{\text{d}}(1 - \phi )}}{(1 - \phi )}$$

where $L_{p}$ is the depolarising factor; $\sigma^{*}$ represents the conductivity of the mixture; $\phi$ represents the porosity of the mixture and the volume fractions of phases 2 ($f_{2}$). Ellipsoidal shape effects are accounted for by introducing the principal depolarization factor, denoted as *L*.

Mendelson and Cohen extended the concept of the depolarization factor to account for ellipsoidal inclusions, providing a detailed analysis of its dependence on the inclusion aspect ratio^54^. The pore shape is defined by the ratio of the principal axes of the ellipsoid. For flattened pore shapes ($\alpha < 1$), $L$ is expressed as follows:

5 $$L = \frac{{1 - e^{2} }}{{2e^{3} }}\left( {{\text{l}} n\frac{1 + e}{{1 - e}} - 2e} \right)$$

where $e = \sqrt {1 - \alpha^{2} }$. For oblong pore shapes($\alpha > 1$), *L* is expressed as follows:

6 $$L = \frac{{1 + e^{2} }}{{e^{3} }}\left( {e - \arctan e} \right)$$

Combining Eqs. (4) to (6), Torquato derived the final form of the electrical DEM theory^55^:

7 $$\frac{{{\text{d}}\sigma^{*} }}{{{\text{d}}\phi }} = \frac{1}{3}\sigma^{*} [\frac{4}{{\sigma^{*} + \sigma_{2} + L(\sigma^{*} - \sigma_{2} )}} + \frac{1}{{\sigma^{*} - L(\sigma^{*} + \sigma_{2} )}}]\frac{{(\sigma_{2} - \sigma^{*} )}}{(1 - \phi )}$$

The electrical DEM model incorporates both the volume fraction and the morphology of inclusions. Analogous to traditional elastic DEM theory, wherein elastic responses are modeled by varying the pore aspect ratio, the electrical DEM framework models resistivity by representing inclusions as geometrically distinct entities. In the context of methane adsorption and desorption in coal, inclusions represent methane-filled pores and microfractures, allowing the model to capture resistivity variations driven by changes in pore structure and methane content.

## Characterization of coal resistivity

Coal rock primarily consists of carbon-rich organic matter and a smaller fraction of inorganic mineral particles. Classification is based on carbon content, calorific value, and the degree of metamorphism of the original plant material, resulting in types such as anthracite, bituminous coal, and lignite. Coal resistivity is influenced by both intrinsic and extrinsic factors. While coal type is a major determinant, resistivity also varies with temperature, pressure, water saturation, and sample heterogeneity. These variables contribute to the broad range observed in resistivity measurements across different studies.

In electrical rock physics modeling, organic matter is treated as a separate phase. Therefore, an accurate estimation of its effective resistivity is essential. Published resistivity data for anthracite, bituminous coal, and lignite under varying temperature, pressure, and saturation conditions were referenced^18,30,38^. Results indicate substantial regional variability in resistivity, with environmental parameters exerting strong control. Table 1 summarizes the statistical characteristics of coal resistivity samples. ‘Std’ refers to standard deviation. The 5th, 25th, 50th, 75th, and 95th percentiles represent the distribution of the sample data.

**Table 1:** Characterization of coal samples.

Data	Clay	Quartz	Feldspar	Calcite	FCad	$R_{{\text{m}}}$	$R_{{\text{c}}}$
Mean	0.768	0.142	0.055	0.035	0.852	2874.200	3149.575
Std	0.023	0.032	0.016	0.011	0.018	74.363	76.011
Min	0.740	0.082	0.029	0.026	0.828	2742.300	3005.800
5%	0.741	0.097	0.033	0.027	0.829	2766.275	3035.095
25%	0.753	0.134	0.048	0.029	0.839	2845.450	3117.250
50%	0.761	0.137	0.057	0.032	0.854	2879.450	3162.250
75%	0.786	0.161	0.062	0.036	0.862	2915.550	3195.050
95%	0.800	0.182	0.074	0.053	0.876	2965.505	3235.685
Max	0.802	0.186	0.079	0.057	0.883	2987.800	3246.500

To estimate the resistivity of the organic phase, the Maxwell–Garnett equation was employed, following the approach by Han et al.^57^ The expression is given as follows:

8 $$\frac{{\sigma_{{{\text{MG}}}} - \sigma_{0} }}{{\sigma_{{{\text{MG}}}} + 2\sigma_{0} }} = \sum\limits_{i} {f_{i} } \left( {\frac{{\sigma_{i} - \sigma_{0} }}{{\sigma_{i} + 2\sigma_{0} }}} \right)$$

where $\sigma_{{{\text{MG}}}}$ represents the conductivity calculated by the Maxwell–Garnett equation; $\sigma_{0}$ and $\sigma_{i}$ represent the conductivity of the background medium and phase *i* medium, respectively. This model is appropriate when inclusions are sparsely distributed and possess conductivities markedly different from the host matrix. Unlike the eHS model, the Maxwell–Garnett approach does not define upper and lower bounds for composite conductivity. Instead, it assumes low inclusion content and uniform dispersion, limiting its applicability in systems with high inclusion fractions or complex geometries.

In Table 1, Clay, Quartz, Feldspar, and Calcite denote the volume fractions of inorganic minerals, while FCad represents the volume fraction of organic matter. $R_{{\text{m}}}$ and $R_{{\text{c}}}$ refer to the resistivity values (Ω·m) calculated using the Maxwell–Garnett equation and the experimentally measured resistivity of coal samples, respectively. The comparison shows strong agreement, with a maximum standard deviation of 0.032 between the modeled and measured resistivities of the organic and inorganic components. The Maxwell–Garnett-derived resistivity for organic matter demonstrates consistency and provides valuable constraints for resistivity modeling in coal.

Variations in Maxwell–Garnett-calculated resistivity are attributed to uncertainties stemming from differences in mineral composition, pore structure, and electrical anisotropy. These uncertainties are considered inherent to the modeling approach. The resulting organic matter resistivity values serve as effective inputs for parameterization in coal electrical rock physics models. This method is applicable to coal from diverse regions and with varying degrees of coalification, enabling reliable estimation of organic resistivity for initial model setup.

Inorganic mineral resistivity spans several orders of magnitude. As reported by Andrade et al.^58^, the selected resistivity values for quartz, feldspar, and calcite are $2 \times 10^{10}$, $4 \times 10^{11}$, and $5 \times 10^{8}$ $\Omega \;{\text{m}}$, respectively, while the resistivity of clay is 1000 $\Omega \;{\text{m}}$. To reduce uncertainty from literature variability, relatively low and uniform resistivity values were adopted for inorganic components.

For methane resistivity modeling, emphasis was placed on simulating changes during the desorption process. Previous studies^34,35^ report that coal resistivity increases approximately linearly with methane desorption, primarily due to reduced gas occupancy in pore spaces. However, the desorption process exhibits hysteresis, with a portion of methane remaining adsorbed even after the process completes. As a result, the post-desorption resistivity shows only a moderate increase.

The initial resistivity during desorption is denoted as $A$ ($\Omega \;{\text{m}}$). A linear relationship is established by curve fitting, with the intercept representing the ideal final resistivity $X$ ($\Omega \cdot {\text{m}}$) after desorption. The methane-related resistivity is expressed as:

9 $$R_{{{\text{CH}}_{4} }} = (X - A)/\phi_{{{\text{coal}}}}$$

where $R_{{{\text{CH}}_{4} }}$ represents the estimated methane resistivity, and $\phi_{{{\text{coal}}}}$ represents the porosity of the coal sample. This expression enables the simulation of dry, water-saturated, and variably coalified samples, offering an effective constraint for modeling methane resistivity in coal.

## Model application

### Resistivity during adsorption and desorption processes

The electrical rock physics model was validated using standard coal samples. The sample had a mass of 600 g, a bulk density of 1.45 g/cm^3^, and a porosity of 5.5%. It remained in an untreated, dried state under ambient conditions throughout the experiment. Before methane adsorption and desorption tests, a calibration of the electrical rock physics model was conducted for the raw coal sample.

The resistivity model incorporated principles of electrical rock physics for analyzing the resistivity of both organic matter and methane in coal. While this framework constrains resistivity values, it does not yield exact measurements. The resistivity of organic and inorganic components differs by several orders of magnitude, making the model sensitive to biases. Therefore, the resistivity of organic matter was first estimated using the Maxwell–Garnett equation. Subsequently, calibration was performed using the average resistivity values of both organic matter and methane.

**Fig. 2:**
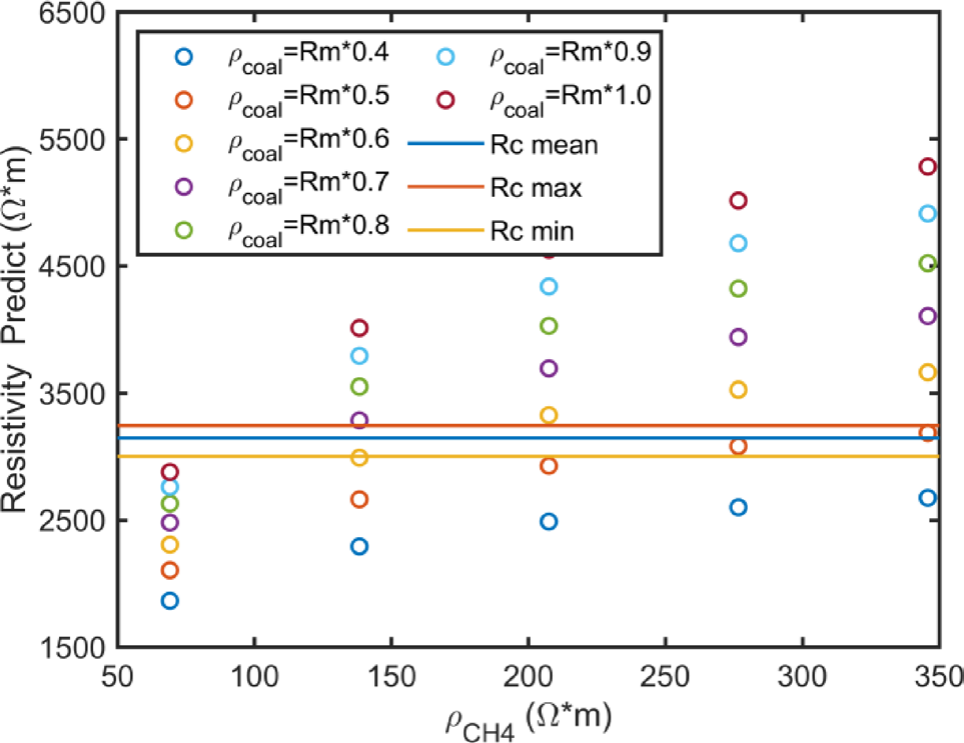
Intersection of predicted resistivity with calibration parameters.

In the eDEM framework, methane is modeled as an inclusion phase. During adsorption, both the aspect ratio and volume fraction of methane inclusions significantly influence the bulk resistivity. Simulation results are presented in Fig. 2. The vertical axis shows coal resistivity values computed using the model, while the horizontal axis represents resistivity parameters after calibration. Calibration coefficients for methane and organic matter resistivity are denoted as $a_{1}$ and $a_{2}$, respectively. The resistivity correction applied in the model is expressed as follows:

10 $$\rho_{{{\text{cal}}}} = a_{i} R_{{{\text{mean}}}}$$

where $R_{{{\text{mean}}}}$ represents the average resistivity of methane or organic material ($R_{{{\text{CH}}_{4} }}$ and $R_{{\text{m}}}$), and $\rho_{{{\text{cal}}}}$ represents the parameter involved in the electrical rock physics modeling calculation ($\rho_{{{\text{CH}}_{4} }}$ and $\rho_{{{\text{coal}}}}$).

Figure 2 shows three horizontal lines representing the mean, maximum, and minimum measured resistivity values of the coal samples. Methane resistivity values ($a_{1}$) are 0.1, 0.2, 0.3, 0.4, and 0.5. For organic material resistivity, the values of $a_{2}$ are 0.4, 0.5, 0.6, 0.7, 0.8, 0.9, and 1.0. Multiple intersections are observed between these model predictions and the measured resistivity ranges. These intersections indicate consistency and can be used to evaluate the suitability of the calibrated parameters within the electrical rock physics model.

After calibrating the resistivity of organic matter, methane, and minerals, the coal sample was used to investigate electrical variations during methane adsorption and desorption. Experiments were conducted at 21 °C and 2 MPa. The corresponding resistivity changes were recorded throughout the process. The pore volume of the coal sample was calculated as the product of its total volume and porosity. The volume of methane occupying the pores during adsorption was then determined using the following expression:

11 $$V_{{{\text{CH}}_{4} }} = \frac{Q\rho }{{m\phi_{{{\text{coal}}}} }}$$

where $V_{{{\text{CH}}_{4} }}$ represents the methane volume fraction, $Q$ represents the methane adsorption amount in the experimental process, $\rho$ and $m$ represent the density and mass of the coal sample, respectively. This calculation yielded the methane volume fraction required for the electrical rock physics modeling.

**Fig. 3:**
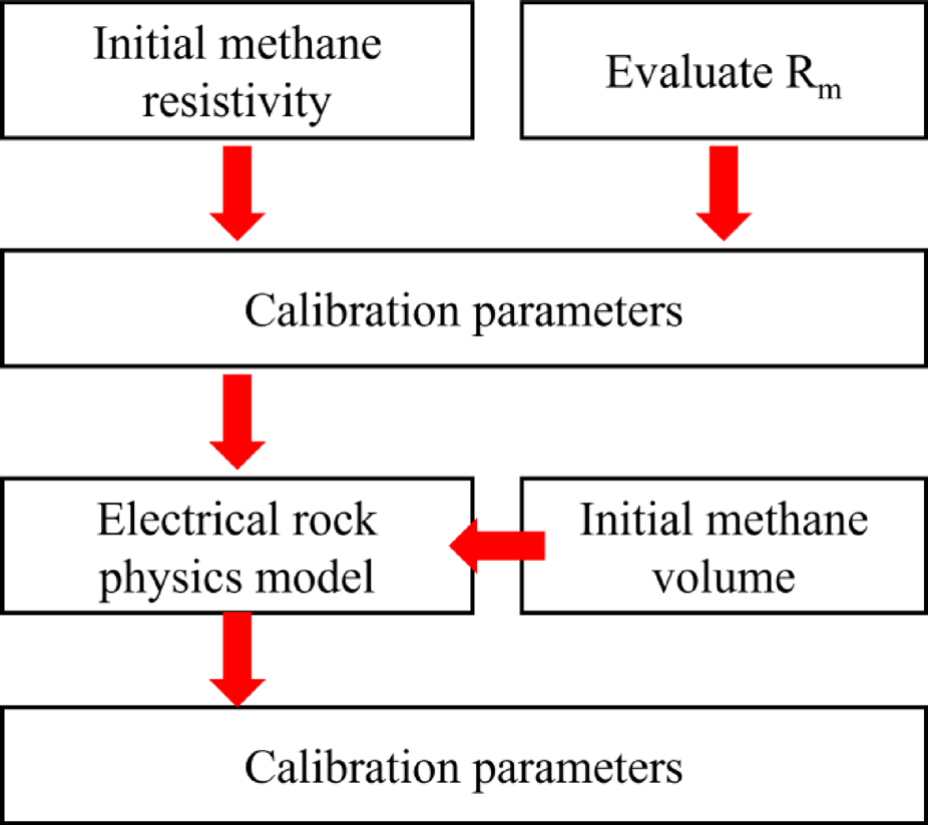
A complete modeling process.

The full modeling workflow is summarized in Fig. 3 and consists of the following steps:

1. Estimation of the initial resistivity of methane using Eq. (9);
2. Using Eq. (8) and Table 1 to evaluate *R*_m_;
3. Evaluation of the calibration parameters using the results of Eq. (10) and Fig. 2;
4. Calculation of methane adsorption from coal rock using Eq. (11);
5. Substituting the above results into the electrical petrophysical modeling process (Modeling Workflow);
6. Evaluation of the effects of methane adsorption on resistivity.

Experimental data were used to establish the relationship between methane adsorption and resistivity. The methane volume fraction was calculated using Eq. (8), and structural effects of the inclusion were incorporated using a traversal optimization method. The inclusion aspect ratio was varied from 0 to 1. Simulation results provided optimal fits, and the best-performing methane and organic matter resistivity correction parameters $a_{1} = 0.2$ and $a_{2} = 0.4$, respectively, were selected.

**Fig. 4:**
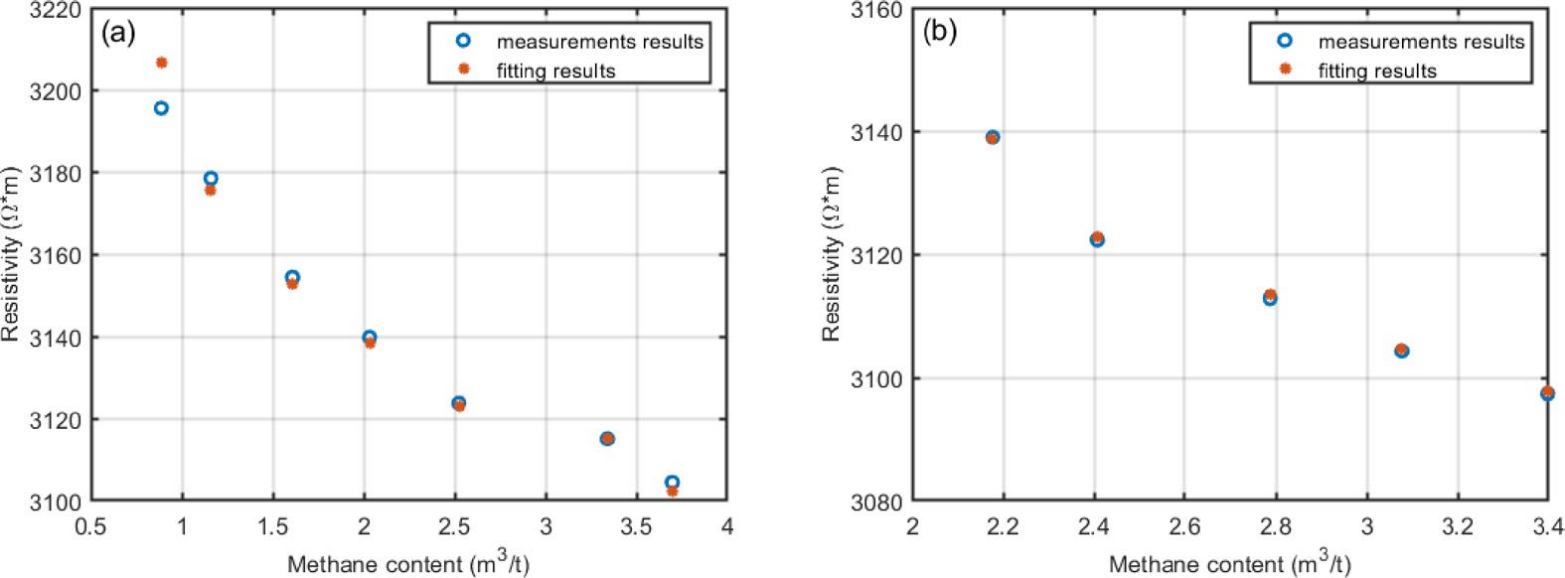
Relationship between methane content during adsorption and desorption to the resistivity of coal samples. (**a**) and (**b**) present the resistivity and electrical rock physics fitting results during the adsorption and desorption of methane in coal samples, respectively.

Figure 4a,b illustrate the relationship between methane content and coal sample resistivity during the adsorption and desorption processes, respectively. The blue scatter points represent the actual measured resistivity of the coal sample, while the orange scatter points represent the resistivity values fitted through the electrical rock physics modeling. As shown in Fig. 4, the resistivity values calculated through electrical rock physics modeling closely match the actual measured values, indicating a strong fitting accuracy.

**Fig. 5:**
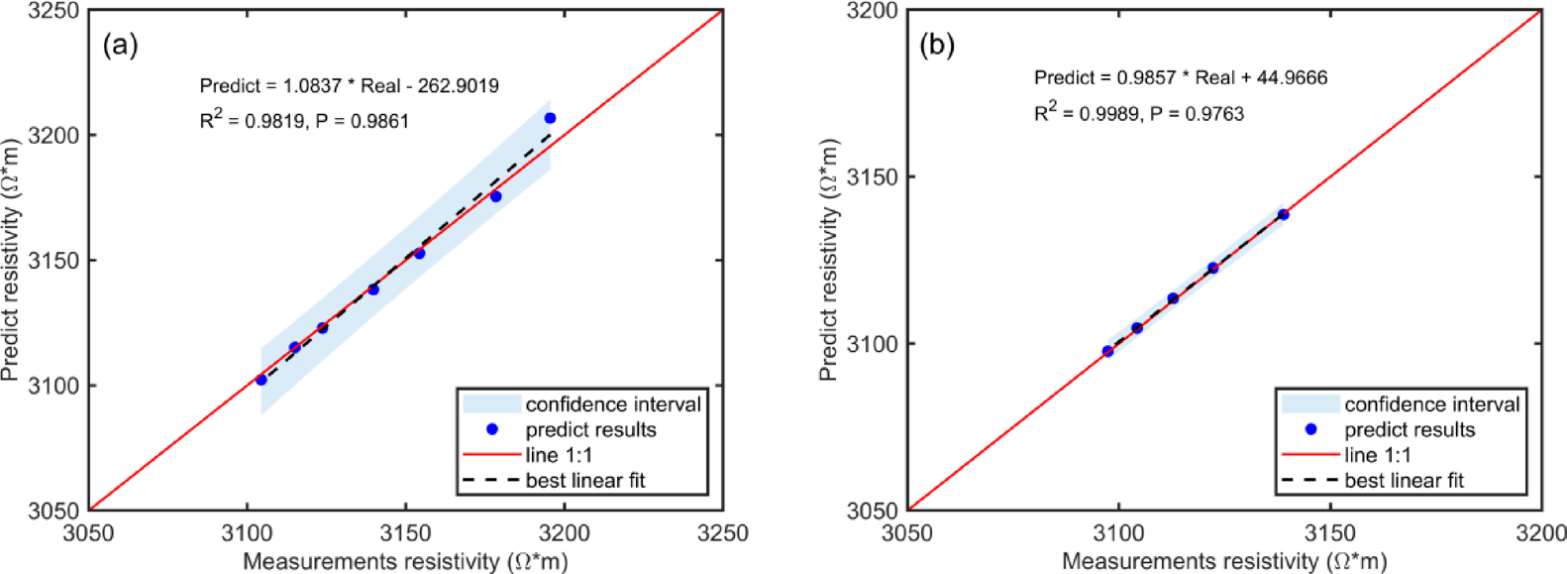
Correlation between experimentally measured and predicted resistivity values. (**a**) and (**b**) represent methane adsorption and desorption, respectively.

Figure 5 illustrates the correlation between experimental and predicted resistivity values. The red line represents the ideal fit, while the blue points denote model outputs. The black dashed line shows the linear regression of the predicted data, and the light blue region indicates the confidence interval. The model achieved R^2^ and P values of 0.9819 and 0.9861, respectively, indicating strong agreement with the experimental measurements. These results confirm that the electrical rock physics model accurately predicts methane-related resistivity changes in coal under the tested conditions.

**Fig. 6:**
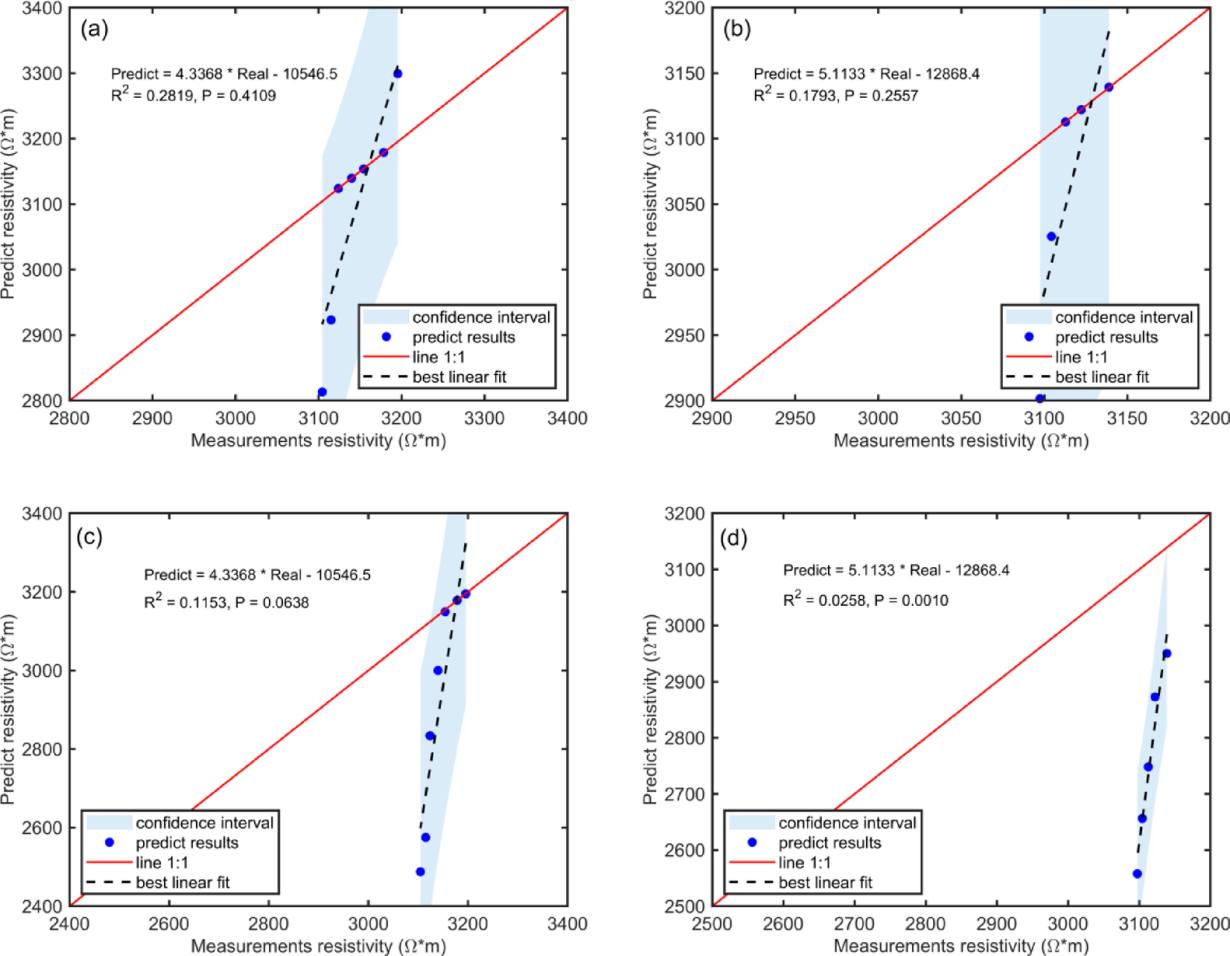
Predicted resistivity of coal samples using different calibration parameters. (**a**) and (**b**) methane and organic matter resistivity correction parameters both set to 0.3. (**c**) and (**d**) methane resistivity correction parameter set to 0.3; organic matter resistivity correction parameter set to 0.2.

Figure 6 presents simulation results for coal sample resistivity using different correction parameters in the electrical rock physics model. In Fig. 6a,b, both the methane resistivity correction parameter and the organic material resistivity correction parameter were set to 0.3. In Fig. 6c,d, the methane resistivity correction parameter was set to $a_{1} = 0.3$, while the organic material resistivity correction parameter was set to $a_{2} = 0.2$. Other parameter combinations were excluded due to poor fitting performance. Figure 6 demonstrates that altering correction parameters for methane and organic matter resistivity decreases the model’s fit quality for adsorption and desorption data, thus reducing its predictive accuracy for coal resistivity. Although some individual data points may be fitted adequately, the low R^2^ values indicate insufficient overall fitting, rendering these parameter sets unsuitable for further study.

Experimental results confirm that methane adsorption significantly influences coal sample resistivity. To quantify this relationship, mathematical fitting (MT) was performed on the experimental data. Quadratic functions were applied to adsorption, and linear functions to desorption processes. Simultaneously, the electrical rock physics model (eRPM) predicted methane adsorption and associated resistivity changes. Table 2 compares the outcomes of MT and eRPM.

**Table 2:** Comparison of eRPM and MT results.

Experimental procedure	Calibration parameters	Fitting method	Fitting formula	R^2^
Adsorption	a1 = 0.2, a2 = 0.4	eRPM	$\rho_{r} = 13.0Q^{2} - 95.4Q + 3271.7$	0.9862
Adsorption	a1 = 0.3, a2 = 0.3	eRPM	$\rho_{r} = - 36.1Q^{2} + 18.9Q + 3255.5$	0.2953
Adsorption	a1 = 0.3, a2 = 0.2	eRPM	$\rho_{r} = - 46.2Q^{2} - 57.9Q + 3307.5$	0.1169
Adsorption	Null	MT	$\rho_{r} = 10.1Q^{2} - 77.3Q + 3254.7$	0.9931
Desorption	a1 = 0.2, a2 = 0.4	eRPM	$\rho_{r} = - 32.14Q + 3204.15$	0.9454
Desorption	a1 = 0.3, a2 = 0.3	eRPM	$\rho_{r} = - 184.1Q + 3569.8$	0.2174
Desorption	a1 = 0.3, a2 = 0.2	eRPM	$\rho_{r} = - 321.8Q + 3648.3$	0.0257
Desorption	Null	MT	$\rho_{r} = - 32.14Q + 3204.14$	0.9473

When appropriate correction parameters are used, methane adsorption and coal resistivity show strong agreement, with similar R^2^ values for adsorption and desorption. This confirms the ability of eRPM to effectively predict methane adsorption and resistivity variations in coal samples. However, deviations in resistivity correction parameters for organic matter and methane lower the R^2^ values, failing to meet accuracy requirements. This limitation highlights the importance of verifying model effectiveness before applying eRPM for reliable resistivity prediction.

### Resistivity of coal components

Electrical rock physics modeling predicts coal resistivity by considering organic matter, minerals, methane adsorption, and coal inclusion structures. This method relies on the electrical and physical properties of coal samples to analyze variations in electrical parameters. The approach enables the prediction of key information for the target area and helps assess how changes in composition and inclusion structures affect coal rock properties, improving understanding of the target region.

**Fig. 7:**
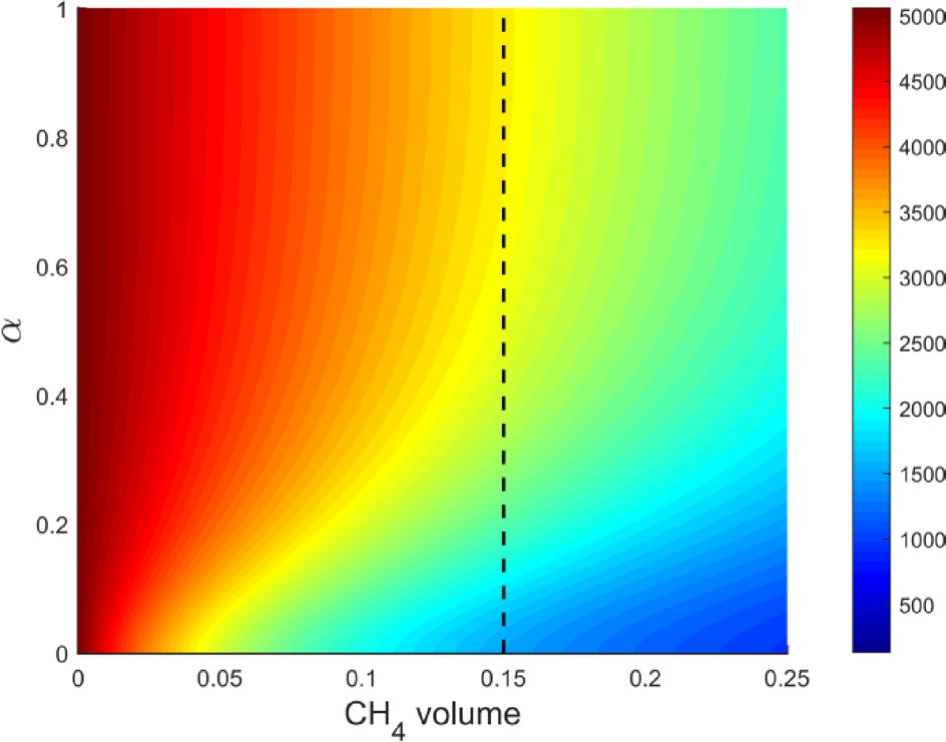
Effect of methane content and inclusion aspect ratio on predicted resistivity.

The mineral composition of the coal samples was analyzed further. The volume fractions of clay, quartz, feldspar, calcite, and organic matter were set to 0.758, 0.137, 0.079, 0.026, and 0.085, respectively. Figure 7 presents predicted resistivity results from electrical rock physics modeling across varying methane volume fractions (0 to 0.25) and inclusion aspect ratios (0 to 1).

Using controlled variable theory, the effects of organic and inorganic mineral components were held constant, isolating the influence of methane adsorption and inclusion structure on resistivity. The resistivity decreased as the methane volume fraction increased, consistent with experimental data. Increasing the inclusion aspect ratio simulated methane adsorbing into the pore structure, transitioning inclusion shapes from flat to ellipsoidal. When methane adsorption stabilized, the flat inclusion structure corresponded to a reduction in coal resistivity. When the methane volume fraction exceeded 0.15 (right side of the black dashed line in Fig. 7), changes in the inclusion aspect ratio had a limited impact on resistivity. Under these conditions, the model’s capacity to represent high-resistivity coal diminished, complicating predictions of methane adsorption effects. This limitation can be attributed to the lower resistivity of methane by several orders of magnitude compared with inorganic minerals and organic matter. Therefore, for high-resistivity coal, accurate representation of organic matter and methane resistivity is critical. Examining intersections between measured coal resistivity and predicted resistivity from the model helps optimize correction parameters for improved modeling accuracy.

In scenarios with a constant inorganic mineral fraction, an increase in organic matter volume fraction corresponds to a proportional decrease in inorganic minerals. Here, the initial organic matter volume fraction was set to 0.75, while clay, quartz, feldspar, and calcite fractions remained at 0.758, 0.137, 0.079, and 0.026, respectively. The methane volume fraction was fixed at 0.1, and the inclusion aspect ratio varied from 0 to 1.

**Fig. 8:**
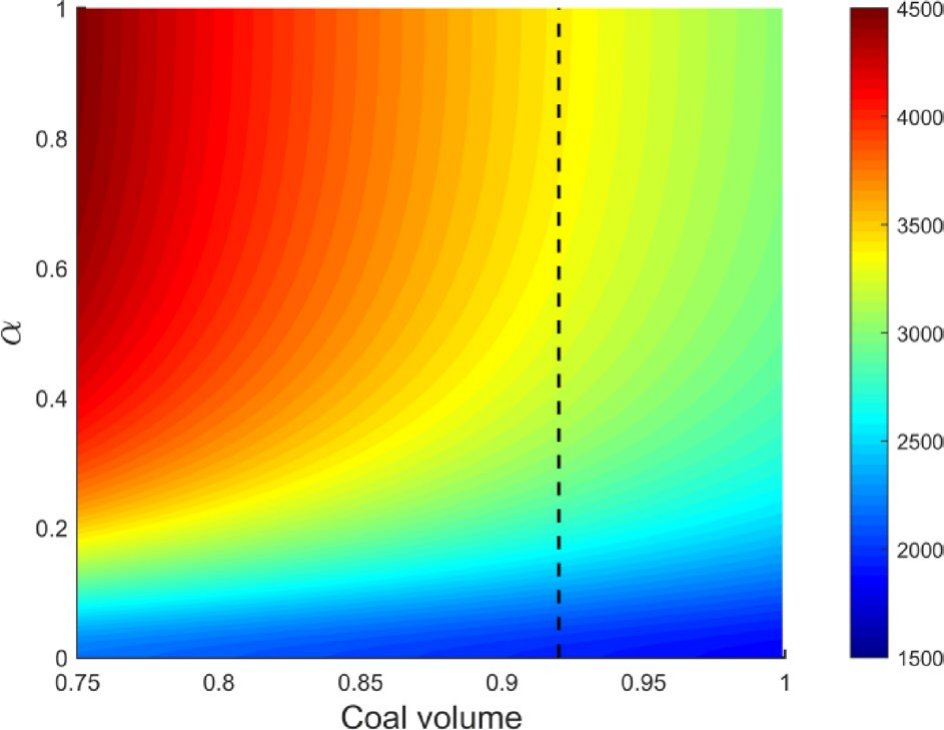
Effect of organic content and inclusion aspect ratio on predicted resistivity.

Figure 8 illustrates the effect of increasing organic matter content on coal rock resistivity. Methane adsorption was fixed, isolating the influence of organic matter, inorganic minerals, and inclusions on resistivity. As illustrated in Fig. 8, resistivity decreases with increasing organic matter content. This trend results from the inherently low resistivity of organic matter and its large volume fraction in coal. The electrical simulation also indicates that resistivity increases with increasing inclusion aspect ratio.

When organic matter content exceeds 0.92 (right side of the black dashed line in Fig. 8), changes in inclusion aspect ratio no longer cause significant electrical variations. At this stage, the model’s ability to represent coal rocks with high organic content weakens. Methane adsorption remained constant in the simulation; thus, the inclusion aspect ratio reflects methane adsorption within pore structures. An aspect ratio lower than 0.1 suggests numerous fractures in the coal rock. An aspect ratio of approximately 0.5 corresponds to the formation of ellipsoidal pores, while values approaching 1 correspond to spherical pore development. Coal with many microfractures exhibits low resistivity, whereas coal with spherical pores shows higher resistivity. Differences in resistivity stem from pore structure and inclusion shape variations.

Detailed experimental analyses through scanning electron microscopy and thin-section observations can refine the range of inclusion aspect ratios. This enables the conversion of experimental findings into numerical simulations and facilitates a deeper exploration of the influence of coal rock pore structure characteristics on resistivity variations.

When effective coal rock data from target exploration areas are available, electrical rock physics modeling can further analyze coal’s electrical properties. This predictive ability surpasses traditional experimental and mathematical fitting methods. Limitations arise from the resistivity values assigned to organic matter and methane, highlighting the need for effective resistivity calibration. Coal resistivity is also affected by temperature, pressure, and water saturation. When experimental data under varying conditions are available, electrical rock physics modeling can evaluate resistivity trends across these parameters, establishing a critical link between coal electrical properties and methane adsorption processes.

## Analysis of model applicability

Key considerations for the applicability of the electrical rock physics modeling workflow include the following:

1. Electrical conductivity in coal rocks involves complex electron and ion movements that are challenging to describe precisely. However, coal resistivity correlates directly with methane content, making its evaluation through electrical rock physics modeling valuable. The model development section details how to assess the resistivity of organic matter, inorganic minerals, and methane required for modeling. This method mainly uses an analytical approach to determine resistivity values for target minerals. In practice, resistivity parameters vary due to mineral composition differences, diagenetic zones, and measurement temperature. Laboratory measurement accuracy and coal sample size significantly affect results. Precise measurements improve analysis quality, while large sample sizes enable better evaluation of organic matter and methane resistivity to approximate effective values. Additionally, employing intersection analysis to calibrate measured coal characteristics with values predicted by electrical rock physics modeling further refines parameter accuracy.
2. Methane filling simulation in coal rocks differs from traditional elastic rock physics modeling. In elastic rock physics, pore structure is evaluated by applying equivalent medium theory to define the rock skeleton, followed by Gassmann’s theory to incorporate fluid effects^59^. By contrast, electrical rock physics modeling does not link pores directly to effective resistivity; resistivity changes arise from charged particle movement within pores. When methane adsorbs into the coal pore structure, water present in coal forms an electrolyte system with minerals. This enables charged particle movement along methane molecules and pore interfaces. The rock skeleton construction is simplified. Methane is introduced into the coal matrix using the eDEM model, assuming it is adsorbed and directly contributes to electrical conduction. Methane is thus modeled as an inclusion in equivalent medium theory. Scanning electron microscopy and other microscopy techniques provide detailed pore and microfracture data to characterize coal pore structures. Modeling can incorporate these pore structure effects following elastic rock physics principles. Laboratory measurements and rock physics analyses calibrate data for coal from different regions.
3. In the modeling workflow, when combining equivalent resistivity values calculated in Steps 1 and 2 using the EHS model, the eHS model is preferred over the eDEM model. The eDEM model adds phases sequentially into the background phase, which does not accurately reflect coal’s diagenetic processes. Methane adsorption aligns better with this simulation approach. Coalification is a continuous, nonlinear transformation over millions of years involving physical and chemical changes. Geological processes create heterogeneity in coal that evolves continuously, complicating precise modeling. This approach simplifies coal evolution to analyze how changes in organic matter, minerals, methane, and pore structures affect resistivity. It offers valuable insights into resistivity variations in coal.

## Conclusions

A dual-coefficient electrical rock physics model was developed to quantitatively link methane adsorption–desorption dynamics with coal resistivity variations, addressing gaps in coalbed methane (CBM) system analysis. The model integrates inorganic mineral composition, organic resistivity, methane adsorption capacity, and pore inclusion structures to simulate resistivity changes during methane adsorption (0.8882–3.6973 m^3^/t) and desorption (3.3974–2.1773 m^3^/t). Key advancements include the introduction of correction coefficients (0.2 for methane, 0.4 for organic resistivity) that reconcile theoretical predictions with experimental data, producing strong correlations (R^2^ = 0.9815 for adsorption, 0.9956 for desorption; *P*-values = 0.9861, 0.9763).

Three principal insights emerge:

1. *Pore geometry dominance*: Inclusion aspect ratios (0–1) strongly influence resistivity. Flattened pores (low aspect ratios) enhance conductivity more than spherical pores, especially at methane volumes < 0.15 m^3^/t.
2. *Organic-mineral interplay*: Organic content inversely correlates with resistivity due to its conductivity. When organic fraction > 0.92, pore structure effects weaken, highlighting mineralogical control over bulk electrical properties.
3. *Adsorption–desorption asymmetry*: Resistivity hysteresis during desorption reflects incomplete methane release, indicating the need for dynamic corrections in gas content estimates.

This model links microscopic adsorption mechanisms with macroscopic resistivity responses, advancing theoretical rock physics and practical CBM management. It offers a scalable framework for optimizing unconventional gas recovery, CO_2_ sequestration monitoring, and coal mine gas hazard prevention. The model adapts to various coal types and structural conditions but requires rigorous calibration of organic and methane resistivity parameters with region-specific validation. Future work should integrate microscopic imaging (e.g., SEM, CT) to refine pore geometry inputs and expand predictive capabilities under multiphysics conditions such as temperature, stress, and fluid saturation.

## Data Availability

Data supporting this study’s results are available from the corresponding author upon reasonable request.

## Latin letters

$f_{1}$: Volume fractions of phases 1 (dimensionless)
$f_{2}$: Volume fractions of phases 2 (dimensionless)
$f_{i}$: Volume fractions of phases *i* (dimensionless)
$L_{p}$: Depolarising factor (dimensionless)
$L$: Principal depolarising factor (dimensionless)
*A*: The initial resistivity of coal during the desorption process ($\Omega \;{\text{m}}$)
*X*: The ideal final resistivity of coal during the desorption process ($\Omega \;{\text{m}}$)
$R_{{\text{m}}}$: The resistivity calculated by Maxwell–Garnett equation ($\Omega \;{\text{m}}$)
$R_{{\text{c}}}$: The resistivity measured of coal samples ($\Omega \;{\text{m}}$)
$R_{{{\text{CH}}_{4} }}$: The estimated methane resistivity ($\Omega \;{\text{m}}$)
$R_{{{\text{mean}}}}$: The average resistivity ($\Omega \;{\text{m}}$)
$a_{1}$: The methane resistivity correction parameter (dimensionless)
$a_{2}$: The organic matter resistivity correction parameter (dimensionless)
$V_{{{\text{CH}}_{4} }}$: The methane volume fraction (dimensionless)
$Q$: The methane adsorption amount (m^3^/t)
$m$: The mass of the coal sample (kg)

## Greek letters

$\sigma_{{{\text{HS}}^{ + } }}$: The upper bounds of conductivity (S/m)
$\sigma_{{{\text{HS}}^{ - } }}$: The lower bounds of conductivity (S/m)
$\sigma_{1}$: Conductivity of phases 1 (S/m)
$\sigma_{2}$: Conductivity of phases 2 (S/m)
$\sigma^{*}$: Conductivity of the mixture (S/m)
$\sigma_{{{\text{MG}}}}$: The conductivity calculated by Maxwell–Garnett equation (S/m)
$\sigma_{0}$: Conductivity of the background medium (S/m)
$\sigma_{i}$: Conductivity of the phase *i* medium (S/m)
$\phi$: Porosity of the mixture (dimensionless)
$\alpha$: Pore aspect ratio (dimensionless)
$\phi_{{{\text{coal}}}}$: Represents the porosity of the coal sample (dimensionless)
$\rho_{{{\text{cal}}}}$: Resistivity for electrical petrophysical modeling ($\Omega \;{\text{m}}$)
$\rho_{{{\text{CH}}_{4} }}$: Resistivity of methane for electrical rock physics modeling ($\Omega \;{\text{m}}$)
$\rho_{{{\text{coal}}}}$: Resistivity of coal for electrical rock physics modeling ($\Omega \;{\text{m}}$)
$\rho_{r}$: The resistivity calculated by electrical rock physics model ($\Omega \;{\text{m}}$)
$\rho$: The density of coal sample (kg/m^3^)
